# A review of attentional bias modification trainings for depression

**DOI:** 10.1111/cns.14022

**Published:** 2022-11-15

**Authors:** Guo Li, Xueli Cai, Qian Yang, Qian Cui, Lihui Huang, Xiujuan Jing, Yifeng Wang

**Affiliations:** ^1^ Psychological Research and Counseling Center Southwest Jiaotong University Chengdu China; ^2^ Institute of Brain and Psychological Sciences Sichuan Normal University Chengdu China; ^3^ School of Public Affairs and Administration University of Electronic Science and Technology of China Chengdu China; ^4^ Tianfu College of Southwestern University of Finance and Economics Chengdu China

**Keywords:** attentional bias modification training, depression, negative attentional bias, rumination

## Abstract

Negative attentional bias is a basic character of depression. The attentional bias modification training (ABMT), being a highly promising and easy‐to‐use depression intervention technique, has attracted much attention to alleviate depressive symptoms in recent years. However, the effectiveness of ABMT programs was mixed across studies, since it remained unclear the underlying mechanisms of ABMT on alleviating depressive symptoms. We systematically analyzed the main ABMT paradigms to clarify possible mechanisms of effective training and reasons of ineffective training. Valid ABMT programs might alleviate depressive symptoms through regulating self‐related rumination or two subcomponents of attentional bias: facilitated attention and impaired attentional disengagement. The reasons for the invalidity of ABMT mainly included the suboptimal design of training procedures, mixed effects of participants' personal characteristics, and the unclear relationship between attentional bias and depression. The ABMT is promising for alleviating depressive symptoms, but training procedures are required to be improved to obtain stable training effects.

## Graphical abstract

The ABMT can alleviate depressive symptoms by the mediation of rumination, by which the involvement of DMN decrease self‐related rumination status. Alternatively, it can also be achieved by changing facilitated attention and difficulty in disengaging, and at the neural level, VAN, and SN are responsible for reducing the orientation to negative stimuli, while DAN and ECN mainly engaged to improve top‐down attentional control and increase attentional disengagement from negative stimuli.

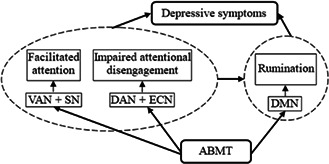

## 1 INTRODUCTION

The major depressive disorder (MDD) is a common mental disability, characterized by persistent low mood, reduced vitality and interest, worse sleep, and high suicidal tendency.^1^, ^2^ It affects more than 350 million people throughout the world.^3^, ^4^ It is predicted that MDD would rank the first cause of disease burden by 2030.^5^

The severe impact of MDD on human beings raised the necessity for its treatment. Accordingly, various therapeutic methods have been applied, including antidepressants, psychotherapy, and physical therapy. Antidepressants were the dominant method in MDD treatment, although it was accompanied by side effects such as insomnia, weight gain, loss of appetite, drug dependence, and other physiological responses that would have dramatic influences on people's daily life.^6^, ^7^, ^8^ Besides that, the uncertainty of which medicine works well for a particular patient took a lot of time, effort, and money for treatment, resulting in unnecessary side effects and low medication adherence.^7^, ^9^ These weaknesses of antidepressants made people turn to other treatments (i.e., psychotherapy and physical therapy). For the psychotherapy, individual psychological counseling, group psychotherapy, and mindfulness were commonly used in the clinical treatment of depression.^10^, ^11^, ^12^, ^13^ Compared with other psychotherapies, the cognitive behavior therapy (CBT) was a widely accepted and effective treatment in alleviating depressive symptoms and reducing the recurrence of depression.^13^ However, the implementation of CBT was usually impeded by the high cost, stringent counseling room, and high requirements of educational level for both consultants and patients. In line with this point, a recent meta‐analysis revealed that only 41% of depressed patients receiving psychotherapy experienced significant alleviation of depressive symptoms.^14^ In terms of the physical therapy, the transcranial magnetic stimulation (TMS) and modified electroconvulsive therapy (MECT) had been confirmed to have significant effects on depression.^15^, ^16^ The side effects of these physical therapies included but not limited to cognitive impairment (especially in memory), discomfort of head and face, facial convulsions, and so on,^7^ which would limit their application. To sum up, the aforementioned methods were useful in treating depression via different ways, whereas their effects were limited in practical application.

Due to these limitations, the computer‐based attentional bias training (ABMT) was put forward based on Baker's cognitive theory.^17^, ^18^ The cognitive theory of depression conceived that people with depression tend to pay more attention to negative than neutral or positive information.^19^ This negative attentional bias, including facilitated attention, difficulty in disengaging, and attentional avoidance,^20^ was deemed as the core problem in depressive individuals^21^, ^22^ and played a vital role in the etiology and maintenance of depression.^23^, ^24^ Specifically, facilitated attention refers to that attention is more easily and quickly drawn by negative information compared with neutral or positive information. Difficulty in disengaging indicates that it would be difficult to switch attention to other stimuli once it has been attracted by negative stimuli. Attentional avoidance refers to that attention is preferentially allocated to other locations rather than the location of negative stimuli. With regard to the three components of negative attentional bias, individuals who are vulnerable to depression tend to focus on negative information and have difficulties in switching attention away from those negative information, thus leading to increased risk of depression.^25^ Therefore, it seems plausible to help people with depression by means of altering their negative attentional bias via ABMT.^26^, ^27^ In line with argument, several studies have recently confirmed that proper ABMTs could alleviate depression to some extent.^26^, ^28^ Compared with other treatments (e.g., antidepressants and psychotherapy), the computer‐based ABMT is easy to operate and has fewer side effects.^29^

Although there were many advantages of ABMT in the treatment of MDD, its effects were quite mixed due to various reasons. Hence, the current study attempted to analyze possible reasons of training effectiveness and ineffectiveness and explore potential mechanisms of ABMT. Additionally, we provided recommendations for developing more effective and professional training programs.

## 2 METHODS

### 2.1 Literature search strategy

We searched Web of Science, CNKI, SpringerLink, PubMed, PsycINFO, Google Scholar, APA PsycNet with the terms depression, depressive, attentional bias, attentional bias modification training (ABMT, ABM), attentional bias training, and cognitive bias modification of attention to obtain relevant literature. The year of publication was restricted up to March 2022.

### 2.2 Literature selection criteria

Selection criteria included: (1) study samples included depressed patients, individuals with residual depression, individuals who have recovered after depression, individuals at high risk of depression, individuals with self‐reported depressive symptoms, other depression‐related groups (these samples must exclude interference from other physical illness or psychological disorders), and normal individuals; (2) ABMT was the only treatment for participants; (3) training procedures were described in detail, including training paradigms, stimuli materials, frequency, location, and number of days of training; (4) training outcomes were measured by depressive symptoms, attentional bias, attentional flexibility, etc., compared between the baseline and post‐training data, or training versus control (placebo group) data; (5) published in English.

### 2.3 Data extraction

Data extraction from eligible studies included sample size, characteristics of participants, intervention and/or control condition characteristics, depression, and attentional bias or attentional flexibility data evaluation.

## 3 RESULTS

### 3.1 Participants

The primary characteristics of included studies are showed in Table 1. Three of the twenty‐one studies recruited depressed patients, two recruited individuals who recovered from depression, one recruited both depressed patients and self‐reported depressed individuals, one recruited both subthreshold depression and non‐depressed females, one recruited girls at familial risk for depression, six recruited only individuals with self‐reported depressive symptoms, and the remaining seven studies recruited healthy individuals to explore whether ABMT changed attentional bias. The total sample size was 1917, including 845 trainees, 670 controls, and 402 without clear subgroup information. The gender ratio was less balanced, with more female subjects. The age ranged from adolescence to older, but the young were predominant.

**TABLE 1:** Overview of characteristics of included studies.

Reference	Participants inclusion	Sample size (gender, mean age)	Training paradigm	Significant result and effect size
30	Experiment 1: undergraduates with depressive symptoms; Experiment 2: depressed patients	*N*1_mildly T_ = 8 (88% female, 19.4 years); *N*1_mildly C_ = 10 (90% female, 19.5 years); *N*1 _moderately‐severely T_ = 17 (94% female, 20.1 years); *N*1 _moderately‐severely C_ = 13 (92% female, 20.5 years) *N*2_T_ = 15 (47% female, 39.9 years); *N*2_C_ = 20 (75% female, 46.3 years).	DPT with word stimuli; complete task every day for 10 days; for training group in 90% of trials probe dot presented in the same locations of positive words.	In training group for mildly depressed sample, score_POMS depression_: pre > post, [*t* (7) = 2.94, *p* = 0.02, *d* = 0.73]; In training group for moderately/severely depressed sample, score_BDI‐II_: pre < post, [*t* (28) = 3.52, *p* = 0.001, *d* = 1.28]; score_POMS depression_: pre < post, [*t* (28) = 2.01, *p* = 0.002, *d* = 1.36]; score_MASQ GDD_: pre < post, [*t* (28) = 3.08, *p* = 0.005, *d* = 1.15]
31	Undergraduate students	*N* = 109 (61% female, 19.0 years)	GAT with faces; complete task only once; asking trainees always focus on the happy faces; only trained a single session.	In training group (SOA = 1250 ms), RT_happy_ < RT_angry_, [*F* (1, 53) = 4.41, *p* = 0.040]; About RT_positive bias_, training group > control group: [*F* (1, 107) = 7.78, *p* = 0.006, η^2^ = 0.07]
32	Unselected adolescents	*N*_EVST_ = 126 (59% female, 14.4 years); *N*_EVST placebo_ = 38 (63% female, 14.4 years); *N*_DPT_ = 128 (56% female, 14.3 years); *N*_DPT placebo_ = 48 (54% female, 14.7 years).	EVST with face pictures; EVST placebo with flower pictures; DPT and DPT placebo with face pictures; in training condition probe stimuli always presented in the same location of happy faces; training lasted 8 weeks, twice a week; first training session was in the laboratory, while the remaining session were at home.	AB for negative information was only found on the EVST [*t* (330) = 10.38, *p* < 0.001, *d* = 0.57], but not on the DPT, [*t* (329) = 1.32, *p* = 0.188, *d* = 0.07]
33	Depressed patients	*N*_T_ = 16 (63% female, 38.3 years); *N*_C_ = 16 (50% female, 39 years).	CTT with face pictures; for training group in 80%–90% of trials cues presented in the same locations of more positive stimuli; training lasted 10 days, once a day.	Compared with the control group, patients in the training group had significantly lower scores at the 1‐month follow‐up: score_BDI_: [(*t* [30] = −5.111, *p* < 0.001)], score_PHQ_: [(*t* [30] = −2.546, *p* = 0.017)], score_HDRS_: [(*t* [30] = −3.758, *p* = 0.001)]. Training group had higher score_AB for happy stimuli_ after training: [*t* (15) = −4.403, *p* = 0.001].
34	University students with self‐reported symptoms of depression	*N* = 40 (75% female, 22.9 years).	12‐min ATT, three‐session intervention.	In training group, score_PHQ‐9_: pre > post > follow‐up, [*p* < 0.001, *d* = 0.53]; In training group, score_DMQ flexibility_: pre < post < follow‐up: [*p* < 0.001, *d* = 0.51]
35	Individuals with depressive symptoms	*N*_T_ = 27 (74% female, 19.4 years); *N*_C_ = 27 (63% female, 19.5 years); *N*_assessment‐only_ = 23 (78% female, 19.6 years).	DPT with word stimuli; for training group in 90% of trials probe dot presented in the same locations of neural words; training lasted for 2 weeks, 8 sessions training totally; post‐assessment included 2‐, 4‐, 8‐week, 3‐ and 7‐month follow‐up assessments.	In training group, score_AB for sad stimuli_: pre > post, [*t* (26) = 8.63, *p* < 0.001, Cohen's *d* = 1.66]; In training group, score_BDI‐II_, pre > post, [*t* (26) = 7.43, *p* < 0.0001, Cohen's *d* = 1.50, 95% CI = 0.90–2.11] The reduction of score_BDI‐II_ from pre‐training to 2‐, 4‐, 8‐week/3‐month follow up, training group > placebo or assessment‐only group: [ts(41–52) > 2.93, ps <0.005, Cohen's *d* = 0.80–2.00]
36	Individuals with at least self‐reported moderate depression severity and negative attentional biases	*N*_AT_ = 48 (73% female, 24.4 years) *N*_ST_ = 49 (78% female, 25.3 years) *N*_NT_ = 48(77% female, 26.1 years)	DPT with face pictures; for active training group in 80% of trials probe presented in the same locations of neural stimuli, while for sham training group only in 50% of trials, and no training group did nothing, except assessing; training lasted 4 weeks, 5 sessions per week.	The reduction of score_QIDS‐SR_, active ABMT > sham ABMT, [*t* = −1.85, *p* = 0.067, *d* = −0.41]; active ABMT > assessment only, [*t* = −2.72, *p* = 0.008, *d* = −0.57] The reduction of score_HDRS_, active ABMT > sham ABMT, [*t* = −2.16, *p* = 0.033, *d* = −0.42]; active ABMT > assessment only, [*t* = −2.28, *p* = 0.021, *d* = −0.49]
26	Patients with a history of Major Depressive Episodes	*N*_T_ = 153 (71% female, 40.2 years); *N*_C_ = 148 (70% female, 41.5 years).	DPT with face pictures; for training group in 83% of trials probe presented in the same locations of more positive stimuli, while for sham training group only in 50% of trials; training lasted 14 days, 2 training sessions per day.	In training group, score_HDRS_: pre > post [*F* (1, 156) = 5.542, η^2^ = 0.03, *p* = 0.02]
37	Unselected adolescents	*N*_T_ = 75 (89% female, 16.4 years) *N*_C_ = 30 (83% female, 16.3 years)	EVST with face pictures; EVST placebo with flower pictures; only trained a single session.	No change in AB between training and control groups: [*F* (1, 94) = 0.04, *p* = 0.85] In training group, score_PANAS_: pre > post [*t* (30) = 2.24, *p* = 0.03, Cohen's *d* = 0.3]
38	Adolescents with major depressive disorder	*N*_T_ = 23 (52% female, 15.09 years); *N*_C_ = 22 (59% female, 14.82 years)	DPT with word stimuli; Two‐stage attention modification procedure: in neutral ABMT, for initial training group in 90% of trials probe dot presented in the same locations of neural words; training lasted for 2 weeks, 8 sessions training totally; in positive ABMT, for booster training group in 67% of trials probe dot presented in the same locations of neural words; training lasted for 2 weeks, 8 sessions training totally post‐assessment included 7‐, 9‐, and 11‐week, and 8‐ and 12‐month follow‐up assessments.	In training group, score_AB for sad stimuli_: pre‐neutral > post‐neutral, [*t* (22) = 6.00, *p* < 0.001, Cohen's *d* = 2.56]; pre‐positive > post‐positive, [*t* (20) = 2.05, *p* = 0.054, Cohen's *d* = 0.92]; In control group, score_AB for sad stimuli_: pre‐neutral > post‐neutral, [*t* (20) = 2.61, *p* = 0.02, Cohen's *d* = 1.16]; pre‐positive < post‐positive, [*t* (15) = 1.19, *p* = 0.25, Cohen's *d* = 0.62]; From pre‐neutral ABM to 7‐week follow‐up, only training group showed reduction (*t* [21] = 6.64, *p* < 0.001, *d* = 2.90) but not the control (*t* [18] =1.72, *p* < 0.05, *d* = 0.81). In training group, depressive symptom counts_,_ pre‐neutral > post‐neutral, [*F* (1, 43) = 5.21, *p* = 0.027, η^2^ = 0.11] In training group, score_HAM‐*D*_, pre‐neutral > post‐neutral, [*F* (1, 43) = 2.83, *p* = 0.10, η^2^ = 0.06]
39	Undergraduate and graduate students	*N*_T_ = 27 (59% female, 22.7 years) *N*_C_ = 26 (62% female, 21.6 years)	DPT with word stimuli; for training group in 94.31% of trials probe dot presented in the same locations of neural words; training lasted approximately 20 min.	In training group, score_AB for sad stimuli_: pre > post [*F* (1, 49) = 5.62, *p* = 0.02]; For score_VAS_, among the high‐dysphoric participants, no difference between the pre‐ and post‐stress in training group, while in control group, pre‐stress < post‐stress; among the low‐dysphoric participants, both training and control group, pre‐stress < post‐stress, [*F* (1, 49) = 4.61, *p* = 0.04]
40	Undergraduates	*N* = 60 (64% female, 20.1 years) There were no differences in age, gender, cognitive vulnerability, and depressive symptoms between training and control group at baseline.	DPT with word stimuli, but presenting a priming stimulus before the presentation of the word pair, for training group in 95% of trials probe dot presented in the same locations of adaptive words, 80 trials	For AB, in training group, participants more likely to attend to adaptive stimuli [*F* (1, 52) = 13.79, *p* < 0.001, *η* ^2^ = 0.21]. For the score_MASQ,_ in training group, participants reported significantly lower levels of depressive symptoms [*F* (1, 23) = 3.61, *p* = 0.07, *η* ^2^ = 0.14].
41	Undergraduates with mild to moderate symptoms of depression	*N* = 30 There were no differences in age score_BDI‐II_ and score_BAI_ between the groups at baseline.	DPT with emotional faces. Six versions of the DPT all differed on two dimensions: direction of training (sad toward neutral or neutral toward happy) and stimulus duration (500 ms, 3000 ms, or variable). For training group in 85% of trials probe dot presented in the same locations of more positive faces; training lasted for 1 week, 4 sessions training totally.	In training group, there were no changes in score_BDI‐II_ and score_BAI_ pre‐ and post‐training, [*Z* = −1.54, *p* = 0.12; *Z* = −1.44, *p* = 0.15].
42	Young and older adults	*N*_young_ = 68 (63% female, 19.4 years) *N*_older_ = 61 (69% female, 72.4 years)	DPT with word stimuli. In positive/negative training, 95% of trials probe dot presented in the same locations of more positive/negative words, 160 trials.	The older received positive training showed the greatest reduction in percent fixations to the most negative areas of the images, [*F* (1, 120) = 5.65, *p* < 0.05, *η* ^2^ = 0.045]. For the young, there were no significant differences in the mood between the pre‐ and post‐training for both positive and negative group, all *p* > 0.54. For the older received positive training, there was no significant difference in the mood between the pre‐ and post‐training, [*t* (56) = 1.80, *p* > 0.05].
27	Girls at familial risk for depression	*N*_T_ = 19 (11.7 years) *N*_C_ = 30 (11.7 years)	DPT with face stimuli. In training group, 100% of trials probe dot presented in the same locations of more positive faces, training lasted for 1 week, 6 sessions training totally (the first session was completed in the laboratory, and the next five daily training were at home).	In training group, decreased attention toward sad faces: pre > post, [*t*_paired_ (18) = −3.51, *p* = 0.002]; increased attention toward happy faces, pre < post, [*t*_paired_ (18) = 2.32, *p* = 0.032]. No significant change in control group.
43	Undergraduate students with mild to moderate symptoms of depression	*N* = 34 (71% female, 19.1 years) There were no differences in age, gender, score_BDI‐II_ and score_BAI_ between training and control group at baseline.	DPT with emotional face and image stimuli; for training group in 85% of trials probe dot presented in the same locations of neural stimuli; training lasted for 2 weeks, 4 sessions training totally, and each training session was approximately 25 min.	For the score_AB for sad stimuli_, the interaction between training condition and time was significant, [*F* (1, 32) = 6.14, *p* = 0.02, *η* ^2^ = 0.16]. In training group, session 1 > session 4, [*t* (15) = 2.16, *p* = 0.047]. In control group, there was no significant difference in score_AB for sad stimuli_ between session 1 and session 4, [*t* (17) = 1.16, *p* = 0.26]. For the depressive symptoms, the interaction between training condition and time was significant, [*F* (2, 31) = 7.31, *p* = 0.003, *η* ^2^ = 0.32]. In training group, baseline > post‐training, [*t* (15) = 3.15, *p* = 0.007, Cohen's *d* = 0.52], baseline > follow‐up [*t* (15) = 4.82, *p* < 0.001, Cohen's *d* = 1.04], and no change was observed in control group for post‐training or follow‐up.
44	Patients with recurrent depression who were not currently depressed	*N*_T‐Faces_ = 16 (62.5% female, 34.6 years) *N*_T‐Words_ = 16 (62.5% female, 40.9 years) *N*_C‐Faces_ = 14 (71.4% female, 37.8 years) *N*_C‐Words_ = 15 (66.7% female, 40.9 years)	DPT with word and face stimuli; for training group in 100% of trials probe dot presented in the same locations of more positive stimuli; training lasted 14 days (28 sessions), 2‐, and 4‐week follow‐up assessments.	The reduction of symptoms, face‐based training group > control group, [*F* (2, 56) = 3.7, *p* = 0.03], whereas no significant effect observed between word‐based training group and control group, [*F* (2, 56) = 1, *p* = 0.4]. The increase of positive bias, face‐based training group, pre‐ < post‐, [*t* (15) = 3.7, *p* = 0.002], whereas no change in the face‐based control group, [*t* (13) = <1, *p* = 0.9].
45	Adults with DSM‐IV Major Depressive Disorder	*N*_T_ = 29 (55.2% female, 28.7 years) *N*_C_ = 23 (60.9% female, 28.1 years)	DPT with face stimuli. In training group, 80% of trials probe dot presented in the same locations of neutral faces, training lasted for 4 week, 8 sessions in‐laboratory training totally, and optional at‐home attention training homework sessions.	In training group, score_AB for sad stimuli_: pre > post, [*z* = −3.62, *p* < 0.001, effect size *r* = 0.45], but not in the control group [*z* = 0.20, *p* = 0.84, effect size *r* = 0.01]. For training group, during the baseline to post, depressive symptoms significantly decreased, [*b* = −1.54, SE = 0.25, *z* = −6.21, *p* < 0.001]; In training group, the change of negative AB was strongly associated with the reduction in symptoms [*r*_p_ = −0.42, *p* = 0.04], but not in the control group [*r*_p_ = −0.05, *p* = 0.83]. And the difference between these correlations were not significant [*z* = −1.34, *p* = 0.18].
28	Clinically depressed individuals	*N*_T_ = 33 (63.6% female, 36.1 years) *N*_C_ = 27 (66.7% female, 34.0 years)	DPT with word and face stimuli; for training group in 100% of trials probe dot presented in the same locations of positive stimuli; training lasted 14 consecutive days, once a day.	After training, the duration of the first fixation on positive words, training group > control group, [*F* (1, 48) = 4.22, *p* = 0.046, *η* ^2^ = 0.081]. After training, total fixation time on positive words, training group > control group, [*F* (1, 48) = 4.37, *p* = 0.042, *η* ^2^ = 0.083]. After training, for the score_CES‐D_, no significant difference between two groups, [*F* (1, 57) = 2.80, *p* = 0.1, *η* ^2^ = 0.047]. After training, for the score_HADS‐A_, training group < control, [*F* (1, 57) = 5.22, *p* < 0.03, *η* ^2^ = 0.084].
46	Subthreshold depression and non‐depressed females	*N*_T_ = 46 (100% female, 20.3 years) *N*_C_ = 26 (100% female, 20.4 years)	DPT with face stimuli. In training group, 87.5% of trials probe dot presented in the same locations of positive faces, training lasted 4 weeks, once a day.	For the score_BDI_, there was a significant time (pre‐, post‐) × group interaction (training, control), [*F* (1, 39) = 20.91, *p* < 0.001]. Post‐hoc comparisons revealed that in the training group, pre‐ > post‐ (*p* < 0.001).
47	Undergraduate students	*N*_T_ = 23 (73.9% female, 20.0 years) *N*_C_ = 17 (64.7% female, 23.5 years)	DPT with face stimuli. In training group, 100% of trials probe dot presented in the same locations of positive faces, while in control group 100% of trials probe dot presented in the same locations of neutral faces, single session, and 320 trials.	For the positive AB, training group > control group, [*F* (1, 36) = 4.71, *p* = 0.036, *η* ^2^ = 0.084]. For the positive mood, there was no effect of training, [*F* (1, 36) = 0.74, *p* = 0.40, *η* ^2^ = 0.07]. For the negative mood, there was no effect of training, [*F* (1, 36) = 0.11, *p* = 0.74, *η* ^2^ = 0.003].

Abbreviations: AB, attentional bias; ABMT, Attentional bias modification training; AT, active training; ATT, attention training task; BAI, Beck Anxiety Inventory. PHQ‐9, The Patient Health Questionnaire‐9; BDI‐II, Beck Depression Inventory‐II; C, control; CES‐D, Center for Epidemiological Studies Depression scale; CTT, cue‐target task; DMQ flexibility, One subscale of Detached Mindfulness Questionnaire used to measure the construct of attentional control/flexibility in ATT studies; DPT, dot probe task; EVST, emotion visual search task; GAT, goal‐directed attention training; HADS‐D, Hospital Anxiety and Depression Scale‐Anxiety; HAM‐D, Hamilton Depression Rating Scale; HDRS, Hamilton Depression Rating Scale; MASQ GDD, One subscale of Mood and Anxiety Symptoms Questionnaire used to measure depression; NT, no training; PANAS, Positive and Negative Affectivity Scale; POMS, Profile of Mood States; QIDS‐SR, self‐reported Quick Inventory of Depressive Symptomatology; RT, reaction time; SOA, stimulus onset asynchrony; ST, sham training; T, training; VAS, visual analog scale used to measure the state of a participant's depressive mood.

### 3.2 Training outcomes

Indicators of training outcomes were varied across studies. Most studies used depression‐related scales (e.g., Hamilton Depression Rating Scale, Beck Depression Inventory‐II, and The Patient Health Questionnaire‐9), while some others used the change of attentional bias score, reaction time to different emotional stimuli, and attentional control/flexibility scales. The results of ABMT were inconsistent across the 21 studies. Specifically, 16 studies relieved depressive symptoms, reduced attentional bias toward negative stimuli, and/or enhanced attentional control after ABMT. One study noted that the old, not the young adults reduced attentional fixation to negative stimuli after positive training. One study showed that ABMT reduced depression mood states for mildly depressed individuals but increased these symptoms for individuals with moderate to severe depressive symptoms. Another study found that the emotional visual search task (EVST) but not the dot‐probe task (DPT) significantly modulated attentional bias. The remaining studies did not show any effect of ABMT.

### 3.3 ABMT paradigms

The modified DPT was the dominant paradigm of ABMT. However, its training effects were unstable. Other paradigms were used less frequently, but all of them were effective in changing attentional bias or alleviating depressive symptoms (see Table 1). Details of these paradigms were described in the following subsections.

#### 3.3.1 The modified DPT

Compared with the traditional DPT, the modified DPT aimed to change participants' attentional bias by directing their attention to the location where it is frequently coupled with positive stimuli. In the modified DPT, a pair of stimuli (e.g., pictures or words) was simultaneously presented for a brief duration (e.g., 500 ms) on the two sides of a fixation cross (i.e., right/left or up/down). The valence of the stimuli was manipulated (e.g., negative–neutral, neutral–positive, negative–positive) according to research objectives. Then, a target (e.g., one or two dots, “:” or “..”, “O” or “Q”, et al.) appeared at either location of previous stimuli. Participants were asked to identify the target by pressing the corresponding button. For the training group, the target would appear at the location where more positive stimulus (i.e., 80% on average) was presented. In contrast, for the control group, positive and negative stimuli were presented equally at either side of the fixation.

#### 3.3.2 The cue‐target task (CTT)

The CTT was mainly involved in the engagement or disengagement of attention.^33^ Specifically, a cue by forms of picture or word would be firstly presented in one of two locations (e.g., right/left), followed by a target stimulus (e.g., “●”) in the same or the opposite location of the cue. Participants were asked to respond to the location of the target by pressing the corresponding button. Similar to the DPT, the cue in CTT could be either emotional or neutral. In order to enhance the positive attentional bias and reduce the negative attentional bias, the targets would appear at the location where more positive cues presented and/or appear at the opposite location where more negative cues presented.

#### 3.3.3 The EVST

Compared with the DPT and CTT that trained participants to unconsciously focus on positive stimuli, the EVST aimed to train participants to actively engage in positive information and disengage from negative information. In the EVST, participants were required to find and select the positive target (e.g., a smiling face) among distracting stimuli characterized by negative valence (e.g., frowning, angry, fearful, or sad faces). For example, the training group was asked to find the smiling face from frowning faces in a 4 × 4 grid, while the control group was asked to find the flower with five petals from flowers with seven petals.

#### 3.3.4 The attention training technique (ATT)

The ATT consists of three training tasks: selective attention, attention switching, and divided attention.^48^ In the selective attention task, participants were asked to focus their attention on specific sounds while disregarding others for about 5 min. In the attention switching task, participants were required to rapidly switch their attention between two types of sounds within 5 min, whereas they were asked to pay attention to multiple sounds at the same time for about 2 min in the divided attention task. It was found that after ATT, the attentional flexibility was improved, and the symptoms of anxiety and depression were reduced significantly. Importantly, these beneficial effects could maintain for 6 months.^34^

#### 3.3.5 The goal‐directed attention training (GAT)

The GAT was developed by Johnson^31^ at the basis of DPT. In the goal‐directed task, the dot appeared at the position where either positive or negative stimulus was presented in the preceding trial. Participants assigned in the training group were required to focus their eyes on the positive stimulus, whereas those assigned in the control group were only asked to complete the DPT. The probabilities of the dot appearing at different locations were the same. Johnson found that, compared with the control group, the training group showed a significant positive attentional bias and reduced frustration response with the stimulus duration of 1250 ms. However, in the training group with stimulus duration of 17 ms and in the control group with stimulus duration of 500 ms, the participants showed angry attentional biases.^31^ This indicated that GAT can enhance subjects' attention bias toward positive stimuli and regulate their emotions.

## 4 DISCUSSION

The ABMT was widely used to help individuals with depression to pay more attention on positive rather than negative stimuli. The conclusions regarding its effectiveness remain controversial. Some studies have shown that individuals with depression significantly reduced their symptoms following the ABMT.^36^ In contrast, others have shown that ABMT has no effect on depressive symptoms.^33^ The reasons for these mixed results would be discussed below.

### 4.1 The mechanisms of valid ABMT

The ABMT was valid in some cases, showing by alleviated depressive symptoms,^39^, ^40^ which were explained by different mechanisms at behavioral and neural levels.

Behavioral, eye‐tracking, and event‐related potentials (ERPs) studies suggested that the effectiveness of ABMTs on depressive symptoms is subject to the processing stage of emotional stimuli. In Johnson's study,^31^ participants showed attentional bias to the positive stimuli when the stimulus onset asynchronies (SOA) was long (i.e., 1250 ms) rather than short (i.e., 17 or 500 ms), implying that the effective impact of ABMT is probably achieved by influencing on the top‐down processing at the later stage of attention. This surmise was supported by a meta‐analysis that attentional maintenance of depressed patients was longer for negative stimuli than positive stimuli.^49^ Specifically, attentional maintenance at the later stage of emotional stimuli processing did differ between clinical patients and healthy controls, whereas attentional orientation occurring at the early stage did not differ between the two groups. However, a systematic review article revealed that not only late ERP components including N2 (related to cognitive control: 250–300 ms) and P2 (related to emotional attention: 200–250 ms) but also early components including error‐related negativity (ERN, related to the error detection: roughly 50 ms post‐error commission) and P1 (related to the early visual processing: 100–130 ms), were affected by ABMTs.^50^, ^51^, ^52^ It seems that ABMTs modulate both the early stage of attentional processing and the later processing associated with cognitive and emotional control. The inconsistency between behavioral and ERP results may stem from the high‐temporal resolution of EEG technique. In other words, the effect of ABMTs on depression shown at the early stage of attentional processing may be hidden in the behavioral data, which can be easily revealed by the ERP technique. In addition to technical factors, these findings were obtained from heterogeneous groups. Because of different baseline brain activities existence in these groups (i.e., depressed patients, subthreshold depressed individuals, high ruminators, and healthy people) before the ABMT,^53^, ^54^, ^55^, ^56^ different processing stages or brain regions might be modulated after similar trainings.

Brain imaging studies uncovered that ABMTs relieved depressive symptoms by modulating specific brain networks and related cognitive functions. Li et al.^46^ observed that the amplitude of low‐frequency fluctuations and functional connectivity in brain regions belonging to the ventral attention network (VAN) became normal after ABMT, suggesting that the VAN and its function in bottom‐up attention was modulated by the ABMT. A recent review on ERPs revealed that ABMTs consistently affected the P2, N2, and ERN.^57^ These ERP components were related to the anterior cingulate cortex (ACC), a key region of the salience network (SN), indicating that the ACC and its role in the detection and orientation of salient stimuli were regulated by the ABMT.

Besides those brain regions responsible for bottom‐up attention, the dorsal attention network (DAN) involved in top‐down attention and the executive control network (ECN) engaged in cognitive control and self‐management of emotion, were also responsible for the alleviation of depressive symptom after ABMT. Rosenbaum et al.^58^ found that high ruminators with defects of inhibition, compared with low ruminators, had lower activation in the DAN under social stress situations. After training with the ATT, activities in the DAN increased compared with the placebo group. Another study of Rosenbaum et al.^59^ by functional near‐infrared spectroscopy (fNIRS) uncovered that attenuated activations in the inferior frontal gyrus (IFG) and dorsolateral prefrontal cortex (dlPFC), core regions of the ECN, in high ruminators were associated with their impaired attentional control. Heeren et al.^60^ reduced the gaze at threatening stimuli in participants with high‐trait anxiety by exerting the transcranial direct current stimulation (tDCS) over the dlPFC during ABMT. This study indicated that the ABMT can modulate the dlPFC and related functions. Taken together, ABMTs could suppress attention to negative stimuli and/or facilitate attention switch away from negative stimuli by restoring brain activities in the VAN, SN, ECN, and DAN. These effects were responsible for the alleviation of depressive symptoms.

It has been suggested that the MDD characterized by negative attentional bias is also closely related to rumination, which refers to repetitive thinking about one's own negative events.^61^, ^62^, ^63^, ^64^ According to the impaired disengagement hypothesis of rumination, when attention cannot disengage from negative information in self‐referential materials, the processing of these materials will be more deeply and detailed.^65^, ^66^ In this perspective, rumination was a typical characteristic of depression and could further exacerbate depressive symptoms.^67^ Yang et al.^35^ found that the ABMT relieved depressive symptoms not only by changing attentional bias directly but also by the mediation effect of rumination indirectly. Neuroimaging studies showed that the self‐referential rumination in depressed individuals led to hyper‐activation in the default mode network (DMN).^68^, ^69^, ^70^ The medial PFC, a hub of the DMN, was demonstrated to be involved in orienting to the biased stimuli by fNIRS.^71^ Therefore, ABMTs could also alleviate depressive symptoms by regulating the DMN and its role in rumination.

**FIGURE 1:**
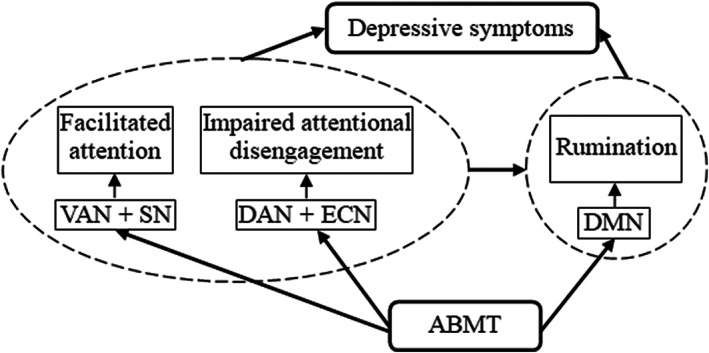
Intervention model of alleviating depressive symptoms by ABMT. DAN, dorsal attention network; DMN, default mode network; ECN, executive control network; SN, salience network; VAN, ventral attention network

The possible mechanisms by ABMTs alleviating depressive symptoms are illustrated in Figure 1. ABMTs mainly modulate two subcomponents of attentional bias: facilitated attention and difficulty in disengaging. They regulate facilitated attention by changing activities in the VAN and SN, thus reducing the orientation to negative stimuli. On the other hand, they modulate difficulty in disengaging by changing activities in the DAN and ECN, thereby improving top‐down attentional control and increasing attentional disengagement from negative stimuli.^45^, ^46^, ^72^, ^73^, ^74^ ABMTs can also alleviate depressive symptoms by reducing activities in the DMN and ruminations of negative self‐referential stimuli. These various mechanisms are associated with multiple training paradigms and diverse characteristics of participants. For instance, the DPT emphasizes the orientation and shifting of attention^75^ while the ATT emphasizes the flexibility of attention control.^34^ In addition, the DMN is specifically modulated in ruminators.

### 4.2 The reasons of invalid ABMT

It is noted that some studies did not find beneficial effect of ABMT on depressive symptoms.^30^ As we mentioned above, the characteristics of participants were an important factor affecting the effect of ABMT. Although negative attentional bias was one of the causes of depression, not all symptoms of depressed individuals were dominated by negative attentional bias.^76^ So the levels of attentional bias prior to the ABMT in different participants might obscure the training effect. Training effects might only be expected when participants showed a negative attentional bias before the ABMT.^36^ In addition, the depression severity might be another participant characteristic that affected training outcomes. Attentional bias was more likely to be changed if the individuals' depressive symptoms were milder.^29^, ^30^ Furthermore, due to the high heterogeneity of depressive symptoms, changing attentional bias without considering other factors was not enough to reduce depressive symptoms.^77^, ^78^ Another notable issue in ABMT studies was the small sample, especially for clinical patients.^30^ To our knowledge, most researches were conducted in the non‐clinical student group, which might not be properly generalized to clinical patients.^30^, ^79^

The design of training programs was another important factor affecting the effect of ABMT. The selection of experimental materials and training parameters, such as training task, type of stimuli (pictures or words), stimulation time (500 ms or 1000 ms, et al.), number of training trials and sessions, interval of sessions, and training strategy, might have variant impacts on the training effect.^26^, ^33^ For instance, de Voogd et al.^32^ used the DPT and the EVST to alleviate participants' depressive symptoms and found that the EVST but not the DPT enhanced the attentional bias to positive stimuli. The low reliability of DPT might be a major cause of inaccurate measurement of attentional bias and insignificant training effect.^80^, ^81^ In addition, even with the same training paradigm (e.g., the DPT), differences in details might produce inconsistent results. In fact, most researches using pictures as stimuli found significant training effects on attentional bias and depressive symptoms, which might be caused by the highly ecological validity.^82^, ^83^ However, the ABMT did not work when using words as stimuli in students with moderate‐to‐severe depressive symptoms and in depressed patients.^30^ Words were more likely to induce self‐referential rumination for depressed participants.^35^ Moreover, the effect of ABMT was more significant for longer durations (i.e., 1500 ms) than shorter durations (i.e., 500 ms) of SOA.^35^ The number of training trials also played a vital role in the ABMT. A systematic, repeated ABMT would produce more effective results than a single training.^82^, ^84^, ^85^ Meanwhile, the longer interval of sessions were the more difficult to maintain the effect of training, which caused the whole ABMT invalid.^35^ Because the ABMT had a slow effect on depression reduction,^33^ the training should last for a long time to stabilize the effect. The training strategy, namely training participants to disengage form negative information or engage in positive information or both of them, was also an important impact factor for the training effect.^26^ In addition to the valence of materials, high‐arousal stimuli could enhance perception and help individuals respond quickly.^86^ In negative–neutral or negative–positive paired scenarios, participants were asked to focus more on high‐valence, low‐arousal neutral stimuli or high‐valence, high‐arousal positive stimuli rather than low‐valence and high‐arousal negative stimuli. However, in a neutral–positive situation, participants were asked to focus more on high‐valence and high‐arousal positive stimuli rather than low‐valence and low‐arousal neutral stimuli. For the latter, it was hard to explain whether the trainee gave priority to high‐valence stimuli or high‐arousal stimuli after the ABMT. In fact, it was more powerful to reduce depression for individuals focusing only on negative self‐referential information.^33^

Last but more importantly, the high probability of positive stimuli as a core parameter of ABMTs might reduce training effects by changing judgment criteria.^87^, ^88^ The initial aim of ABMT is to train individuals to shift their attention to positive stimuli, while the high probability of positive stimuli and low probability of negative stimuli might make participants tend to evaluate ambiguous and less negative stimuli as negative, coupled with the fact that one of the characteristics of depression is the negative interpretation of ambiguous information.^29^ Therefore, depressive symptoms might be mitigated and aggravated simultaneously, weakening the training effect or even worsening the symptoms.

### 4.3 Limitations

The limitations of this study were as follows. Firstly, key information was missing in some included studies, such as participants' information, details of training procedures, and the effect size of training, so the results needed to be interpreted with caution. Secondly, as the sample size of clinically depressive patients was small, the estimation of ABMT on the alleviation of depressive symptoms might be affected. Finally, these studies evaluated the effect of training with different criteria (e.g., modification of attention bias, alleviation of depressive symptoms, improvement of attention flexibility, and others), which might affect the comparisons of training effects among studies. Therefore, there was a great demand for a larger sample size of clinical‐‐depressed individuals, more training details, and uniform rating criteria to assess the alleviation of depression by ABMT.

## 5 FUTURE DIRECTION

In order to get greater benefits from ABMT, some researchers have paid more attention to improve training procedures. Firstly, the eye‐tracking based ABMT (ET‐ABMT) was developed^89^ to overcome the low reliability of classical reaction time‐based ABMT.^90^ With the eye‐tracking technique, what kind of stimuli caught participant's attention first or more attention, as well as other valuable information, could be continuously assessed. These data could provide supportive evidence for the effect of ABMT, making outcomes more diverse and reliable.

Secondly, immediate feedback was provided to promote the learning process of the trainee. For instance, the Eye‐gaze contingent attention training (ECAT)^91^ required participants to unscramble a scrambled sentence into a positive self‐statement as quickly as possible (e.g., “am a winner I loser born” → “I am a born winner”) and pay attention to positive words. After that, participants received the online instant gaze‐contingent feedback (e.g., You looked 67% of the time at the positive words) on their attention allocation while unscrambling the sentence. In the reward‐based, eye‐tracking ABMT paradigm,^92^ one happy face and three sad faces were randomly arranged in a 2 × 2 grid on the computer screen. Participants freely viewed the pictures but were asked to pay attention to the ones that became clearer. At the same time, the positive images that were blurred gradually became clearer as the participants gaze at them longer, while the negative images remained blurred. In the gaze‐contingent music reward therapy (GC‐MRT),^93^ participants listened to their wanted music only when they fixed on the positive pictures. These paradigms linking attention to positive stimuli during training with enjoyable experiences (e.g., clear pictures, favorite music) possibly led the depressed individuals actively to be engaged in the training, which could then facilitate the training effect more or less.

Thirdly, much work has been done to optimize program parameters. Appropriate trials, sessions, and intervals of sessions were explored.^35^ Training that approximates daily situations were suggested to improve the transfer effect.^94^ Self‐referential materials were also suggested to improve the training effect.^33^ Many other efforts have been paid to improve the effectiveness of ABMT, making it a highly promising, easy‐to‐use depression intervention technique.

In addition to improving existing paradigms, it is necessary to develop new ABMT paradigms based on attentional theories. For instance, the rhythm theory of attention suggests that attentional sampling is a cyclical fluctuation processing at the theta rhythm. Attention tends to stay on the current target at the peak of the theta rhythm but shift to other stimuli at the trough.^95^, ^96^ The trough of the theta rhythm, therefore, may serve as a promising training paradigm or parameter for the shifting of attention.

## 6 CONCLUSION

The ABMT is a cognitive‐based depression intervention technique, which is a promising treatment for depression since it is easy to operate and has few side effects. However, the unreasonable design of training procedure, ambiguous characteristics of trainees, and unclear mechanisms of intervention might lead to ineffective ABMT. Therefore, standardized and effective ABMT programs are still being explored. Cognitive and neural mechanisms of effective ABMT are the core problems to be solved in future investigations.

## AUTHOR CONTRIBUTIONS

GL and XC wrote the first draft and revised the manuscript. QY were instrumental in its improvement. QC, LH and XJ contributed to reviewing and editing. YW provided invaluable guidance throughout its preparation and approved the final version of the manuscript. All authors contributed to the article and approved the submitted version.

## FUNDING INFORMATION

This work was supported by the National Nature Science Foundation of China [grant number 62177035, 31700947]; Sichuan Province Social Science Planning Project [grant number 20JY175]; Science and Technology Department Project of Sichuan Province [grant number 2022NSFSC1505]; “Talent Teacher Training Program‐Green Seedling Plan” of Southwest Jiaotong University.

Role of the Funding Source. The funders of the study had no role in study design, data collection, data analysis, data interpretation, or writing of the report. The corresponding author had full access to all the data in the study and had final responsibility for the decision to submit for publication.

## CONFLICT OF INTEREST

None.

## Data Availability

Data sharing not applicable to this article as no datasets were generated or analysed during the current study.
